# In Vitro Model for Simulating Drug Delivery during Balloon-Occluded Transarterial Chemoembolization

**DOI:** 10.3390/biology10121341

**Published:** 2021-12-16

**Authors:** Jorge Aramburu, Raúl Antón, Junichi Fukamizu, Daiki Nozawa, Makoto Takahashi, Kouji Ozaki, Juan Carlos Ramos, Bruno Sangro, José Ignacio Bilbao, Kosuke Tomita, Tomohiro Matsumoto, Terumitsu Hasebe

**Affiliations:** 1 Tecnun Escuela de Ingeniería, Universidad de Navarra, 20018 Donostia-San Sebastián, Spain; ranton@tecnun.es (R.A.); jcramos@tecnun.es (J.C.R.); 2 IdiSNA, Instituto de Investigación Sanitaria de Navarra, 31008 Pamplona, Spain; bsangro@unav.es (B.S.); jibilbao@unav.es (J.I.B.); 3 Terumo Corporation, 3-20-2, Nishi-Shinjuku, Shinjuku-ku, Tokyo 163-1450, Japan; junichi_fukamizu@terumo.co.jp (J.F.); daiki_nozawa@terumo.co.jp (D.N.); 4 Terumo Medical Pranex, 1500 Inokuchi, Nakai, Ashigarakami 259-0151, Japan; makoto_takahashi@terumo.co.jp (M.T.); kouji_ozaki@terumo.co.jp (K.O.); 5 Liver Unit and CIBEREHD, Clínica Universidad de Navarra, 31008 Pamplona, Spain; 6 Department of Radiology, Clínica Universidad de Navarra, 31008 Pamplona, Spain; 7 Department of Radiology, Tokai University Hachioji Hospital, Tokai University School of Medicine, 1838 Ishikawa-machi, Hachioji, Tokyo 192-0032, Japan; ktomita@tsc.u-tokai.ac.jp (K.T.); t-matsu@kochi-u.ac.jp (T.M.); hasebe@tokai-u.jp (T.H.)

**Keywords:** liver cancer, in vitro modeling, hemodynamics, B-TACE, microballoon catheter

## Abstract

**Simple Summary:**

Liver cancer is one of the leading causes of cancer-related deaths worldwide and balloon-occluded transarterial chemoembolization (B-TACE) has emerged as a safe and effective treatment for liver cancer. However, the hemodynamic alterations that are responsible for the successfulness of the treatment and are produced by the microballoon catheter used during the treatment are not yet well understood. In this study, we developed an in vitro model (IVM) that can simulate B-TACE. We designed clinically relevant experiments, and we obtained clinically realistic results. We conclude that the IVM allows for a visual understanding of a complex phenomenon (i.e., the blood flow redistribution after balloon occlusion) and it could be used as a base for future sophisticated and even patient-specific IVMs; in addition, it could be used to conduct IVM-based research on B-TACE.

**Abstract:**

Background: Balloon-occluded transarterial chemoembolization (B-TACE) has emerged as a safe and effective procedure for patients with liver cancer, which is one of the deadliest types of cancer worldwide. B-TACE consist of the transcatheter intraarterial infusion of chemotherapeutic agents, followed by embolizing particles, and it is performed with a microballoon catheter that temporarily occludes a hepatic artery. B-TACE relies on the blood flow redistribution promoted by the balloon-occlusion. However, flow redistribution phenomenon is not yet well understood. Methods: This study aims to present a simple in vitro model (IVM) where B-TACE can be simulated. Results: By visually analyzing the results of various clinically-realistic experiments, the IVM allows for the understanding of balloon-occlusion-related hemodynamic changes and the importance of the occlusion site. Conclusion: The IVM can be used as an educational tool to help clinicians better understand B-TACE treatments. This IVM could also serve as a base for a more sophisticated IVM to be used as a research tool.

## 1. Introduction

Transarterial chemoembolization (TACE) is used to treat patients with primary liver cancer [1], which is the third leading cause of cancer deaths in 2018 [2]. Conventional TACE consists of the intraarterial infusion of anticancer agents mixed with lipiodol followed by occluding particles that reduce blood flow and induce tumor necrosis [3]. TACE is the standard of care for patients with intermediate hepatocellular carcinoma [4], although Facciorusso et al. [5] reported no superiority of TACE over transarterial embolization (TAE). 

A novel form of TACE, the balloon-occluded TACE (B-TACE), was recently reported by Irie et al. [6]. B-TACE is performed with a microballoon catheter that temporarily occludes the feeding arteries. Compared to conventional TACE, it has been reported that B-TACE can improve lipiodol accumulation in tumors, especially when the balloon-occluded arterial stump pressure (BOASP) (i.e., the pressure distal to the catheter tip) is below 64 mmHg [6,7,8].

Once the balloon occludes the feeding arteries, an upstream and a downstream compartment are created, and these remain connected via collateral arterial circulation, which determines the value of BOASP [9]. These collateral pathways are crucial for the supply of the biliary tract [10]. Examples of these pathways include the communicating arcade (CA) that connects the right hepatic artery (RHA) and left hepatic artery (LHA) [11], which are also connected to the 3-o’clock and 9-o’clock arteries that feed the common bile duct [10], i.e., the pathways of the peribiliary plexus around the intrahepatic bile ducts [12]. The hilar plexus can also provide connections between the different segments of the liver [10]. However, it is often difficult to visualize the collateral flow in medical images and, therefore, understand the importance of it.

The liver is an organ with a unique dual blood supply that includes many extra- and intrahepatic collateral vessels [10,13]. Extrahepatic collaterals enter the liver parenchyma from nonhiliar entrances; for example, the phrenic artery gives extrahepatic afferences that enter through the nude area. Intrahepatic collaterals deliver blood to the hepatic sinusoids (or hepatocites or tumors) from vessels that may replace this function when the main afferences (i.e., lobar, segmental or subsegmental arteries) are occluded for any reason. 

These collateral vessels have been used to allow blood flow redistribution after artery balloon occlusion. Applications ranged from tumor detection [13,14] to tumor treatment [9,15]. Currently, collateral circulation plays a pivotal role in microballoon-catheter-assisted endovascular procedures [16,17]. After balloon occlusion, the upstream and downstream compartments remain connected via the collateral vessels, and the BOASP does not reduce to zero [9], meaning that collateral pathways that are not always visible under X-ray images exist.

In the case of B-TACE, the blood flow persists after the temporary occlusion of a hepatic artery owing to the existing intrahepatic and extrahepatic collateral pathways. The hepatic artery mean pressure drops from a pressure before occlusion of *P*_bo_ ≈ 90 mmHg to a value known as BOASP (or pressure after occlusion, i.e., *P*_ao_), which could be used to assess the suitability of a patient for a successful B-TACE: If BOASP is greater than a threshold pressure (i.e., *P*_thres_), then collaterality is high and the pressure distal to the occlusion site does not change considerably. Under this setting, the benefits induced by the balloon occlusion (i.e., protection against retrograde lipiodol delivery and preferential targeting of tumors) might be lost [9].

**Figure 1:**
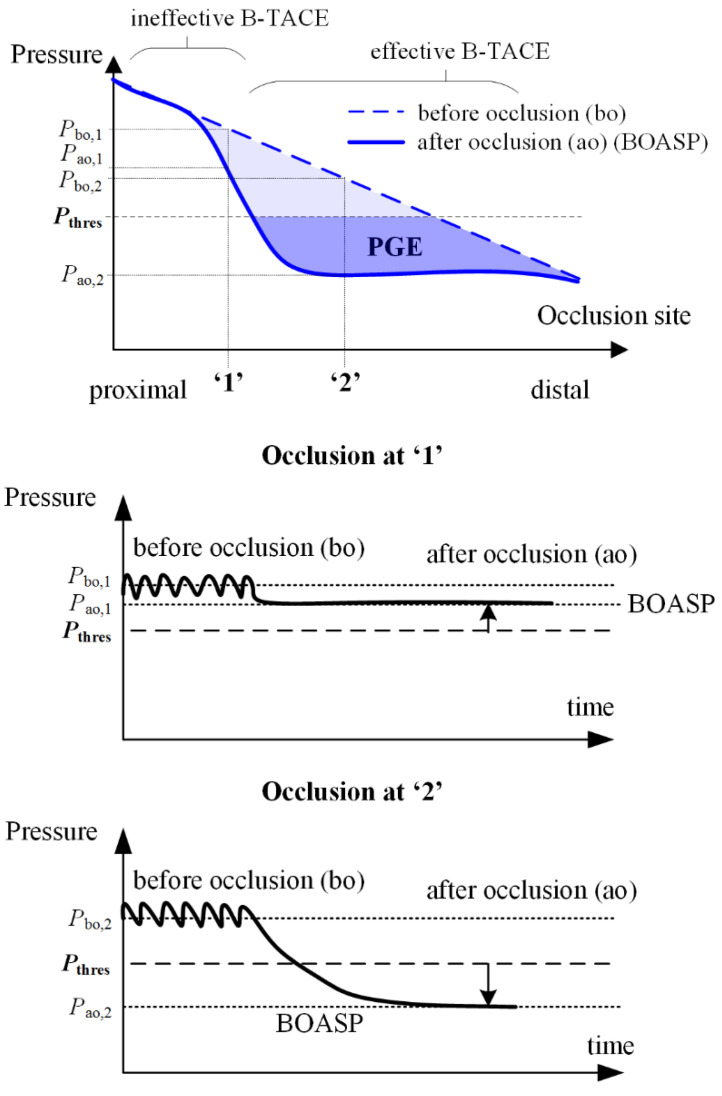
On top, the conceptual relationship between the occlusion site in the hepatic artery (from proximal to distal locations) and the average blood pressure before and after the occlusion. For two occlusions at ‘1’ and ‘2’, the blood pressure before and after the occlusion is depicted in time. Proximal occlusions result in ineffective B-TACE, and more distal occlusions result in effective B-TACE [18]. The hepatic artery pulsatile pressure before occlusion turns into a reduced constant pressure after balloon occlusion. The average arterial pressure before occlusion is typically *P*_bo_ ≈ 90 mmHg. The pressure after occlusion *P*_ao_, (i.e., BOASP) determines whether the B-TACE is successful. If *P*_ao_ is greater than a threshold pressure *P*_thres_, *P*_ao_ > *P*_thres_, then the pressure-gradient effect (PGE) is not promoted, and B-TACE is unsuccessful. If *P*_ao_ < *P*_thres_, then PGE is promoted, and B-TACE is successful. Irie et al. [6] reported a value of 64 mmHg for *P*_thres_. The subscripts ‘bo’ and ‘ao’ mean before occlusion and after occlusion, respectively.

In contrast, when BOASP goes below *P*_thres_, the pressure distal to the balloon changes considerably and effectively (Figure 1). Irie et al. found that *P*_thres_ = 64 mmHg [6]. Under this second scenario, a pressure gradient between distal normal- and tumor-tissues is promoted, and the pressure-gradient effect (PGE) ensures a preferential tumor-tissue irrigation to redistribute blood flow from normal to tumor tissues through collateral pathways [6]. PGE during B-TACE enhances lipiodol accumulation in tumor tissues and reduces nontarget embolization in normal tissues. In a later study, Matsumoto et al. [18] analyzed the influence of the occlusion site, identifying microballoon locations that may result in effective or ineffective B-TACE.

Modeling has shown the potential to analyze the influence of treatment parameters (catheter type, infusion site, infusion velocity, etc.) and even optimize other transcatheter intraarterial treatments, like radioembolization [19,20], and modeling could also help to understand the collateral circulation in the liver during B-TACE. Recently, Aramburu et al. [21,22] analyzed B-TACE via numerical simulations of hemodynamics and developed a hepatic artery model that includes collateral arteries. The model provides the platform to study hemodynamic changes in various scenarios. 

The representative model of hepatic artery studied by Aramburu et al. [22] was replicated in the in vitro model (IVM) development in this study. Bioengineered IVMs are an invaluable research tool for better understanding of tumor microenvironments and testing anticancer therapies [23,24]. To the best of our knowledge, there is no IVM of the hepatic artery hemodynamics available that incorporates collateral circulation or replicates the promotion of collateral flow redistribution when a microballoon catheter is inflated during B-TACE. The aim of this study is to present a simple IVM that replicates a complex phenomenon, i.e., an IVM of the arterial hemodynamics of the liver that includes collateral circulation, to visually study changes in collateral circulation.

## 2. Materials and Methods

In this study, we first developed the conceptual design of the IVM. Then, we created the mathematical model of the IVM. The purpose of this mathematical model was twofold: it was used to perform a simulation-based sizing of the geometrical characteristics of the IVM (e.g., the height of reservoirs, the length and diameter of tubes, etc.) to ensure that physiologically realistic flowrates flowed through the IVM, and it was also used to perform simulation-based quantitative analyses of pressures and flows in the IVM, which were used to complement the qualitative results observed in the experiments. 

Based on the simulation-based sizing, the actual IVM was built. On this IVM, several experiments were carried out, in which qualitative analyses were performed. We examined the influence of the location of the tip and the cancer scenario via a series of experiments. Finally, we compared the results of the experiments with two clinical cases, and we saw that these cases are similar to two of the experiments we conducted, suggesting that the conceptual design was appropriate.

### 2.1. Conceptual IVM Design

The main objective was to demonstrate PGE during B-TACE in an IVM that permits the visualization of hemodynamic changes due to the collateral circulation in the liver. The hepatic arterial flow is in vitro modeled with a hydraulic system that captures all the important hemodynamic features during B-TACE (e.g., collateral pathways that permit collateral circulation) and is able to replicate PGE (i.e., flow from normal to tumor tissues under appropriate balloon occlusion). 

A real-scale in vitro hepatic artery model is included in the IVM to allow for realistic B-TACE in vitro experiments with actual microballoon catheters. For simplicity, steady-state operation of the IVM is adopted, water is used (instead of blood), and dyed-water is injected (instead of chemo-lipiodol emulsion). Therefore, it is difficult to obtain physiologically realistic pulsatile flowrate (mL/min) and pressure (mmHg) values in the hydraulic system. In the IVM, the treatment outcome is regarded as successful when PGE is developed and when the injected agent is directed only to tumor-feeding branches.

**Figure 2:**
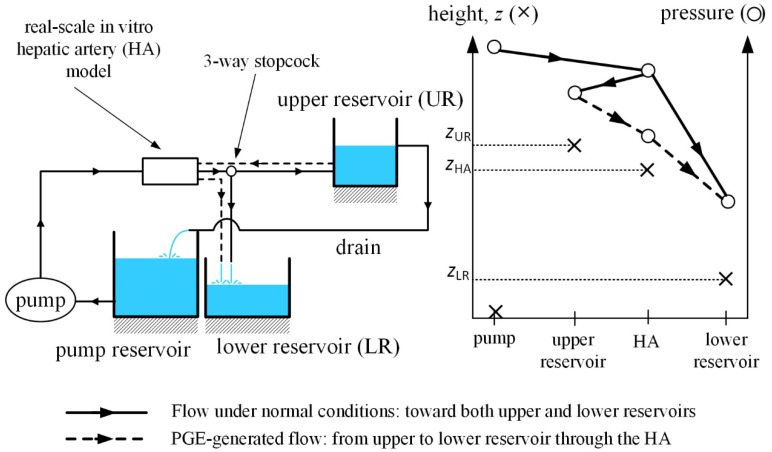
Conceptual design of the IVM. A schematic is shown on the left. The pump generates the water flow and feeds the real-scale hepatic artery in vitro model. Under normal (i.e., no occlusion) conditions, represented by the continuous line, water is directed either to the healthy-tissue-modeling upper reservoir or to the tumor-tissue-modeling lower reservoir. Under an occlusion scenario, the pump-generated flow can persist or stop. If the pump-generated flow persists, then both reservoirs remain being fed. If the pump-generated flow stops, then the PGE-generated flow is promoted, represented by the dashed line, and the flow is redirected in the direction from the upper to the lower reservoir, passing through the hepatic artery. The constant free-water surface level in the upper reservoir is ensured by draining water from the upper reservoir to the pump reservoir. The figure on the right shows the actual heights and pressures at the pump, the upper reservoir, the hepatic artery and the lower reservoir. Flow is driven by pressure differences. Under normal conditions or under occlusion conditions where the pump-generated flow persists, the pressure at the hepatic artery is greater than that of the reservoirs, and thus flow is in the direction of the reservoirs (continuous line). When pump-generated flow is stopped, the flow is driven by the differences in the height of the free-surface level of the upper and lower reservoirs, which are greater and smaller than the height of the hepatic artery, respectively. Therefore, the flow is in the direction from the upper to the lower reservoir, passing through the hepatic artery.

The IVM models the whole systemic circulation, which is reduced to a simple hydraulic system. Figure 2 shows the schematics of the system, which consists of three functional reservoirs, a pump, a real-scale in vitro hepatic artery, three-way stopcocks and tubing connecting the parts. Water is irrigated through a pump toward the real-scale in vitro hepatic artery model. The hepatic artery model is horizontally located and consists of a symmetric bifurcating structure with a single inlet and various outlets, with collateral pathways allowing flow between branches at the same level. Each of those outlets is connected to a three-way stopcock, by which the characteristic of the outlet is defined: it feeds either tumor tissue or normal tissue. 

If an outlet feeds tumor tissue, the flow is directed toward the lower reservoir, which models tumor-tissue pressure. In contrast, if an outlet feeds normal tissue, the flow is directed toward the upper reservoir, which models normal-tissue pressure. Water in the upper reservoir is drained to the pump reservoir, modeling the venous flow, and the pump pumps the water from venous pressure level to hepatic artery pressure level. Under steady-state operation, the free surface of water in the upper reservoir remains unchanged, and the lower reservoir is filled under the presence of tumors. 

The IVM must replicate the hepatic arterial hemodynamics during B-TACE. Under normal conditions, water must flow from the pump toward upper or lower reservoirs through normal-tissue-feeding or tumor-feeding outlets after flowing through the real-scale in vitro hepatic artery model. 

To do so, the pressure provided by the pump must be greater than the pressure of water in both the upper and lower reservoirs. When simulating B-TACE by occluding a branch in the hepatic artery, two scenarios can arise. First, when the flow through the pump is interrupted and PGE promotes collateral circulation from upper to lower reservoir through the hepatic artery. In this case, the physiological normal-to-tumor tissue-pressure gradient is replicated with a gravity-driven flow. To do so, the height of the free surface of water in the upper reservoir (*z*_UR_, Figure 2) must be greater than that of the lower reservoir (*z*_LR_, Figure 2), and the height of the hepatic artery model (*z*_HA_, Figure 2) must be between the heights of the water free-surfaces of the reservoirs (i.e., *z*_UR_ < *z*_HA_ < *z*_LR_). 

Second, when flow through the pump is not interrupted because of a main-flow redirection through collateral arteries and water flows toward upper and lower reservoirs as under normal conditions. Figure 2 shows the heights and water pressures of the pump, upper reservoir, hepatic artery model, and lower reservoirs. Lines and dashed lines indicate the flow directions.

### 2.2. Mathematical Modeling and IVM Sizing

In order to size the hydraulic system depicted in Figure 2, a zero-dimensional (0D) model of fluid dynamics was used. In addition to using the mathematical to size the geometric characteristics of the IVM, this model was used to complement the qualitative results of experiments, by simulating the same experiments numerically and providing quantitative flow and pressure results.

**Figure 3:**
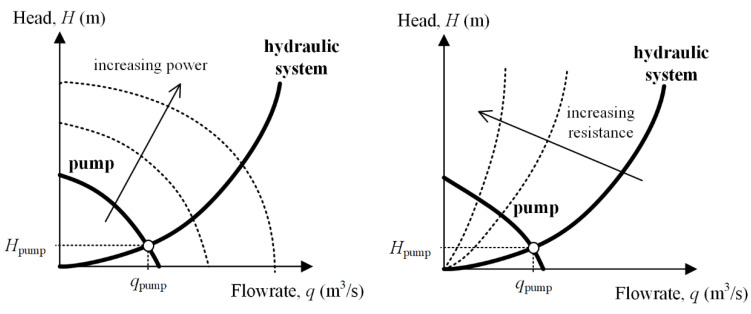
Head–flowrate relations for hydraulic systems.

The system consists of a pump, a real-scale hepatic artery model, an upper reservoir, a lower reservoir, a pump reservoir and tubing, and three-way stopcocks that connect the hepatic artery to the reservoirs. The pump must provide the system with sufficient head (i.e., energy per unit weight) to overcome the hydraulic resistance of the system and replicate the flow toward the hepatic artery and upper or lower reservoirs. If the pump is started and its power is gradually increased, the flowrate increases; if the hydraulic resistance of the system increases, the flowrate provided by the pump decreases. For a given hydraulic system and a given pump, the working point corresponds to the intersection of the pump curve and the characteristic curve of the system (Figure 3).

In a single circular and straight tube where a Newtonian fluid flows in steady state, the following equations apply:

(1) Δp=Rq

(2) $$R=\frac{128\mu }{\pi }\frac{l}{d^{4}}$$

where Δp (Pa) is the pressure difference between the two ends of the tube, R (Pa·s/m^3^) is the hydraulic resistance, q (m^3^/s) is the volumetric flowrate, μ (Pa·s) is the dynamic viscosity of the fluid, and l (m) and d (m) are the length and the diameter of the tube, respectively.

**Figure 4:**
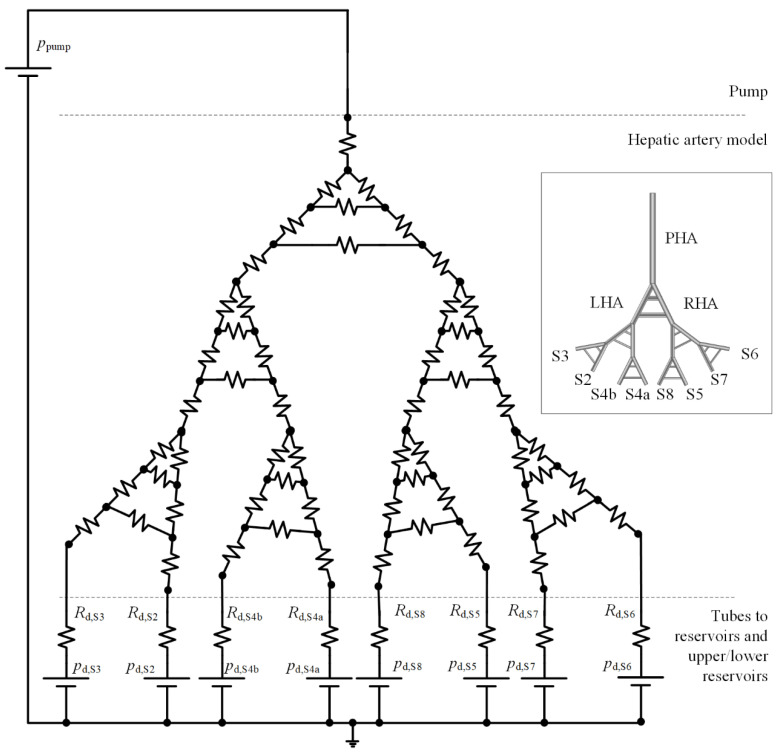
Zero-dimensional model of the IVM. *R_d_* and *p_d_* indicate the downstream resistance and pressure, respectively.

The IVM can be reduced to a 0D model consisting of a set of branches connected by nodes. By using the fluid–electric analogy, the pump is modeled as a constant-voltage source, the hepatic artery and tubes are modeled as resistances, and reservoirs are modeled as constant-voltage sources (Figure 4).

The hepatic artery resistances are calculated with Equation (2) with the length and diameters of branches known from Aramburu et al. [22]. To define the remaining parameters of the 0D model, seven parameters must be sized via numerical simulations to meet the specifications in conceptual design and design criteria of easy set-up, transport, and manipulation of the IVM:

- Length of tubes connecting outlets of the hepatic artery and lower reservoir, *l*_LR_ (m).
- Diameter of tubes connecting outlets of the hepatic artery and lower reservoir, *d*_LR_ (m).
- Length of tubes connecting outlets of the hepatic artery and upper reservoir, *l*_UR_ (m).
- Diameter of tubes connecting outlets of the hepatic artery and upper reservoir, *d*_UR_ (m).
- Height of the free surface of water in the lower reservoir, *z*_LR_ (m).
- Height of the free surface of water in the upper reservoir, *z*_UR_ (m).
- Head (energy per unit weight) provided by the pump, *H*_pump_ (m).

Downstream resistances (*R_d_* in Figure 4) are calculated with Equation (2), and downstream constant-pressure sources (*p_d_* in Figure 4) and pump pressure (*p*_pump_ in Figure 4) are calculated with Equations (3) and (4), respectively, where *ρ* is the water density (1000 kg/m^3^) and *g* = 9.8 m/s^2^.

(3) $$p_{d}=\rho gz_{d}$$

(4) $$p_{\mathrm{pump}}=\rho gH$$

If Kirchhoff’s laws are applied to the system, a system of linear equations with the following form is obtained:

(5) [R]{q}={p}

where [R] is a matrix that contains resistances, {q} is a vector containing flowrates, {p} is a vector containing pressure values. Equations (5) was solved as explained in Aramburu et al. [22].

The cancer scenarios that are defined in Section 2.4 were used for sizing. For each case, three catheter locations were assumed: conventional TACE (i.e., no balloon occlusion), B-TACE with the microballoon at location 1, and B-TACE with microballoon at location 2. By manually adjusting the sizing parameters, the following values were found to be adequate for a proper demonstration of PGE and appropriate to implement in the IVM: $l_{d,\mathrm{LR}}$ = 40 mm, $d_{d,\mathrm{LR}}$ = 1.2 mm, $l_{d,\mathrm{UR}}$ = 40 mm, $d_{d,\mathrm{UR}}$ = 1.2 mm, $z_{\mathrm{LR}}$ = 80 mm, $z_{\mathrm{UR}}$ = 110 mm, and $H_{\mathrm{pump}}$ = 200 mm.

**Figure 5:**
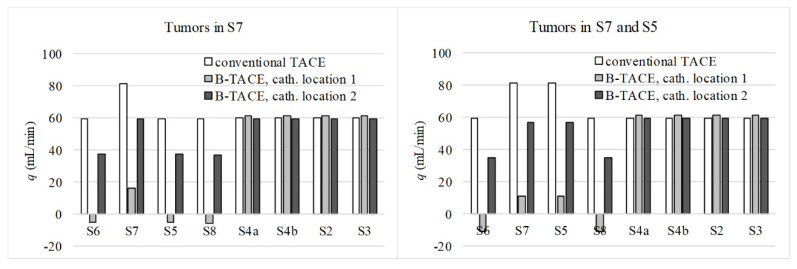
The results of numerical simulations for IVM sizing.

Figure 5 shows the results for the two cases (i.e., tumors in S7 and tumors in S5 and S7) and three catheter locations (no balloon occlusion, catheter location 1, and catheter location 2). The results are qualitatively the same for the two cases. Focusing on the case with tumors in S7, the reported combination of sizing values results in physiologically realistic flow distributions. With no occlusion, a greater amount of blood flows toward the tumor-bearing segment S7 compared to the other segments. 

Under occlusion at location 1, PGE-driven flow is promoted from S6, S5, and S8 (negative flowrates) toward S7, resulting in a successful treatment. With the occlusion at location 2, flow redirects through a collateral pathway and flow distribution in the right lobe is similar to the distribution during conventional TACE. However, the flowrate values decrease due to the increase in the hydraulic resistance to flow in the collateral pathway. These results suggest a successful sizing of the IVM.

### 2.3. Actual IVM Design

**Figure 6:**
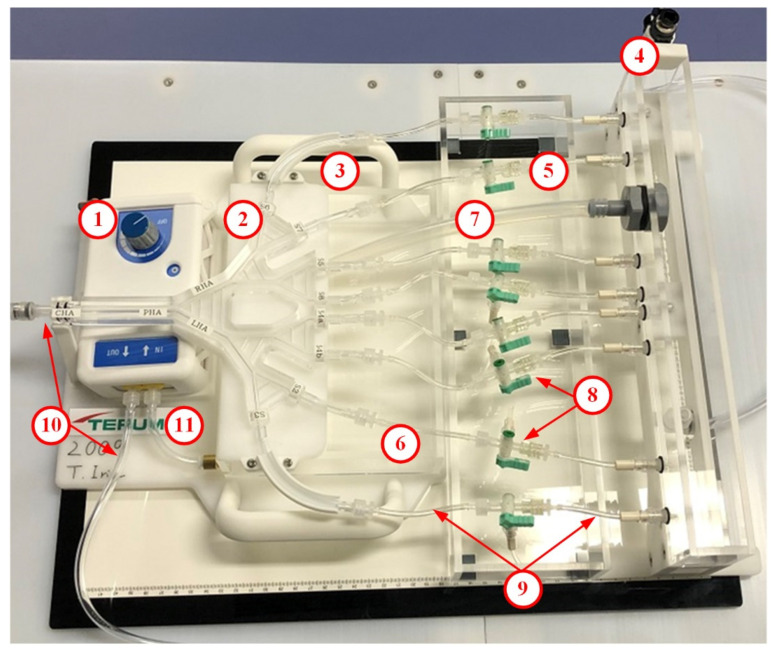
The actual IVM that consists of the (1) the irrigation pump, (2) the real-scale in vitro hepatic artery model, (3) the dock and panel, (4) the upper reservoir, (5) the lower reservoir, (6) the pump reservoir, (7) the outflow drain, (8) the three-way stopcocks, and (9–11) the tubing connecting the parts.

The design objectives of the IVM are easy set-up, operational stability, educational scenarios, and in vitro PGE performance. The IVM (manufactured by Terumo Corporation, Tokyo, Japan) fulfills the design requirement to enable easy transport in the dedicated carrying case, easy set-up in 20 min with minimal 500 cc of water supply, and stable manipulation of complex tubing, stopcocks and the pump including stable flow distribution in the arterial model. The parts of the IVM are shown and explained in Figure 6 and Table 1, respectively. For practical reasons, the length and diameters of the tubes in the IVM differ slightly from those in the preliminary numerical simulation-based sizing shown in the previous section; however, the qualitative results (PGE promotion after a proper balloon occlusion) are the same.

**Figure 7:**
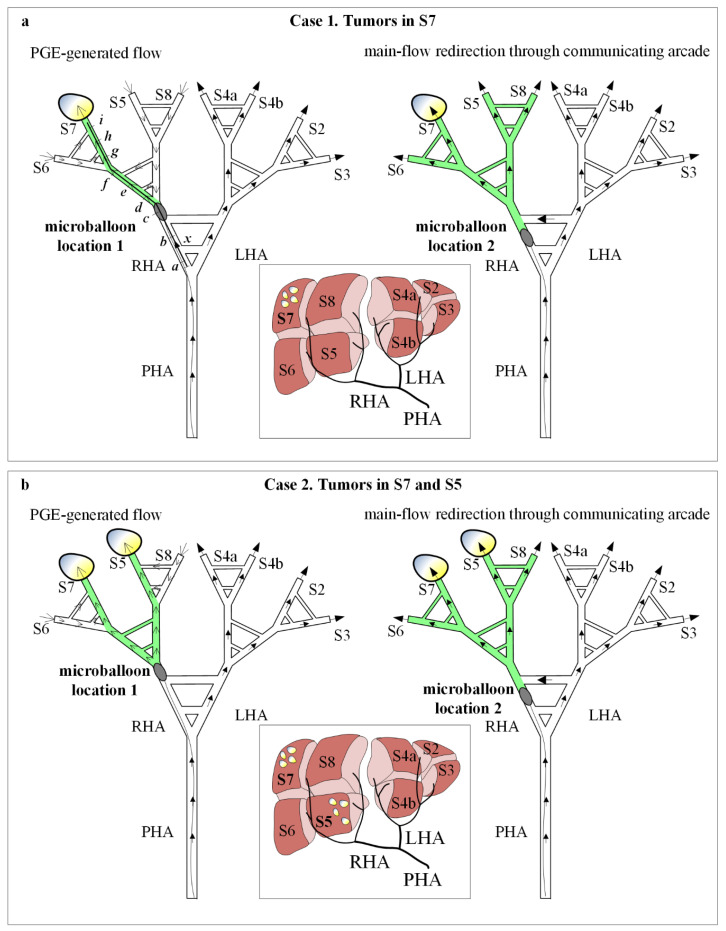
Cases and microballoon locations in experiments. (**a**) In case 1 (tumors in S7) with microballoon location 1 (Experiment 1), PGE promotes collateral flow (open arrows) toward tumors. Microballoon locations ‘*a*’ to ‘*i*’ are defined for Experiment 5. In case 1 with microballoon location 2 (Experiment 2), blood flow is redirected through the communicating arcade (filled arrows) and treatment is directed toward the whole right lobe. In case 2 (tumors in S7 and S5) with microballoon location 1 (Experiment 3), PGE promotes collateral flow (open arrows) toward tumors. (**b**) In case 2 with microballoon location 2 (Experiment 4), blood flow is redirected through the communicating arcade (filled arrows) and treatment is directed toward the whole right lobe.

The hepatic artery model follows the model designed by Aramburu et al. [22] See (2) in Figure 6 to see the actual IVM and Figure 7 to see the branching pattern and the outlet nomenclature. This hepatic artery consists of the proper hepatic artery (PHA), which bifurcates into the RHA and LHA, each of which bifurcate until eight segmental arteries are generated, with segments (S2, S3, S4a, S4b, S5, S6, S7, and S8) defined according to Couinaud’s classification [25]. This morphology is the most common one among the hepatic artery configurations [26,27]. 

It is important to note that the hepatic artery includes the following collateral pathways: connections between the RHA and the LHA modeling the CA [28], connections between branches of the same level of generation modeling the intersegmental connections via the hilar plexus [10] or can be seen as the connections when there are watershed tumors that share more than one feeding artery [29].

Regarding educational scenarios and in vitro PGE performance, variables that users can operate in the IVM are (i) the location of the microballoon catheter and (ii) the location of tumor tissue and healthy tissue, which can be controlled by the state of the three-way stopcock at each outlet. The steps to prepare the IVM and catheters prior to the demonstration of educational scenarios and in vitro PGE performance include the following:

- To set-up the IVM by connecting tubing, stopcocks, a pump, and electricity cables.
- A camera that transmits live high-resolution video on a screen can be used to simulate the fluoroscopic anterior-posterior projections.
- To define the educational scenario to identify the segment with tumors by placing the three-way stopcocks at the desired direction.
- To pour water to the pump reservoir and the upper reservoir and start the pump until steady-state flow conditions are obtained.
- To introduce the microballoon catheter to the desired location. In this study, the followings are used: a 2.7-F Occlusafe^®^ (Terumo Corporation) for a microballoon catheter, a 4-F Glidacath^®^ (Terumo Corporation) for a guiding catheter, and a 0.016-in Radifocus^®^ GT-wire (Terumo Corporation) for a microwire.
- To test the flow distribution by the injection of dyed-water fluid (representing chemo-lipiodol emulsion) and adjust the pump speed.
- To demonstrate educational scenarios of combination of the location of the microballoon catheter and the tumor and healthy tissues.
- To replace the water in the reservoirs when the transparency is lost due to repeated injections of dyed water fluid.

### 2.4. IVM Testing

To test the IVM the two user-controllable variables were used: two cancer scenarios and two catheter locations. Case 1 corresponds to tumors in S7 and Case 2 corresponds to tumors in S7 and S5; microballoon location 1 corresponds to the RHA after the pathways that model the CA, and microballoon location 2 corresponds to the RHA between the pathways that model the CA (Figure 7). Regarding the definition of cancer scenarios, it is important to note that this version of the IVM allows us to consider a liver segment to be either a tumor-bearing segment or a normal segment, by connecting the outlet to the lower reservoir or upper reservoir, respectively. 

Therefore, the presence of tumors in a segment is modeled with a segment having a lower pressure compared to that of a normal segment; therefore, the location of the nodules within the segment is not considered. Cases and microballoon locations were combined to define four experiments. In addition, a fifth experiment was defined with one of the cases and multiple proximal-to-distal microballoon locations to analyze when PGE develops and B-TACE is successful. This fifth experiment was also performed numerically, using the mathematical model used during the IVM sizing to support the qualitative results with quantitative results. 

Moreover, according to the conceptual IVM design in Section 2.1, the results of the experiments can be foreseen by analyzing the influence of the microballoon location on the flow in the specific in vitro hepatic artery of the IVM after balloon occlusion.

#### 2.4.1. Experiment 1

Experiment 1 consists of the cancer scenario case 1 (i.e., tumors in S7) with microballoon location 1 (Figure 7a left). Under these conditions, the main arterial flow (filled arrows) toward the right lobe stops and collateral circulation is promoted in the direction of the tumor-bearing S7 (open arrows). The resulting B-TACE treatment is successful in redistributing the treatment agent toward tumors.

#### 2.4.2. Experiment 2

Experiment 2 consists of the cancer scenario case 1 with microballoon location 2 (Figure 7a right). Under these conditions, main arterial flow (filled arrows) toward the right lobe persists because it redirects through the CA. The resulting B-TACE treatment is unsuccessful because treatment is distributed toward both healthy and tumor tissue.

#### 2.4.3. Experiment 3

Experiment 3 consists of the cancer scenario case 2 with microballoon location 1 (Figure 7b left). As in Experiment 1, the main arterial flow (filled arrows) toward the right lobe stops and collateral circulation is promoted in the direction of the tumor-bearing S7 and S5 (open arrows). The resulting B-TACE treatment is successful in redistributing the treatment agent toward tumors.

#### 2.4.4. Experiment 4

Experiment 4 consists of the cancer scenario case 2 with microballoon location 2 (Figure 7b right). As in Experiment 2, the main arterial flow (filled arrows) toward the right lobe persists because it redirects through the CA. The resulting B-TACE treatment is unsuccessful because treatment is distributed toward both healthy and tumor tissue.

#### 2.4.5. Experiment 5

Experiment 5 consists of the cancer scenario 1 with multiple microballoon locations (i.e., ‘*a*’ to ‘*i*’, Figure 7a left). Indeed, Experiment 1 and Experiment 2 are part of this experiment, where Experiment 1 is Experiment 5 with the microballoon at ‘*c*’, and Experiment 2 is Experiment 5 with the microballoon at ‘*b*’. This experiment was conducted to analyze the microballoon locations that promote PGE.

Experiments 1–5 were recorded (see Supplementary Materials). In addition, Experiments 1 and 2 were recorded with red-colored water in the upper reservoir to visualize how PGE develops.

#### 2.4.6. Numerical Simulations of Experiment 5

In addition to the multiple-location in vitro Experiment 5, numerical simulations of this experiment were carried out using the model explained in Section 2.2 to support the qualitative experimental results with the quantitative numerical results.

### 2.5. Clinical Data

**Figure 8:**
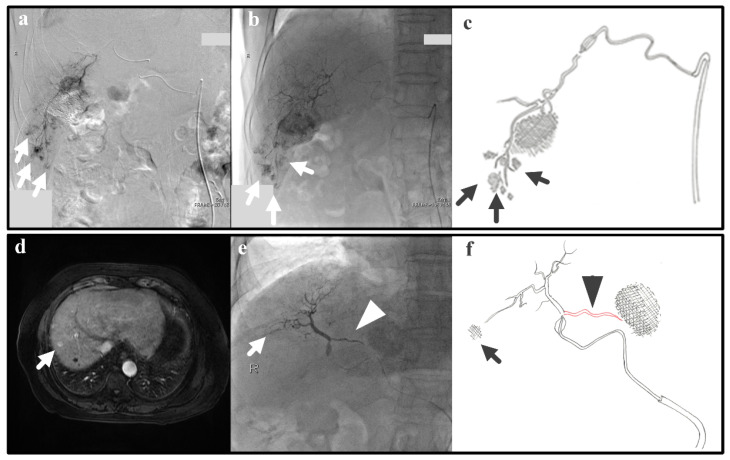
Patient 1: (**a**) Angiogram for tumor detection, (**b**) lipiodol accumulation during B-TACE, (**c**) a hand drawing showing the tumors, the hepatic artery, and the microballoon catheter. Patient 2: (**d**) MRI for tumor detection and (**e**) no lipiodol accumulation in the tumor (arrow), collateral pathway promotion (arrowhead) during B-TACE, and (**f**) a hand drawing showing the target tumors, the hepatic artery, the microballoon catheter, and the collateral artery.

In real clinical cases, arterial hemodynamics is complex due to varied and not-always-visible collateral pathways. Thus, we examine real cases that are relevant to the IVM testing; despite the simplicity of the IVM, the successfulness/unsuccessfulness of clinical cases can also be explained with the IVM. The following two clinical cases represent Experiments 1 and 2, which give as a result efficient and inefficient B-TACE procedures, respectively. Experiment 1 (i.e., a single tumor and successful B-TACE) is similar to the case of a patient (patient 1) with previous embolization of a 3-cm tumor in S6 that underwent B-TACE after multiple early stains were detected in angiogram (arrows in Figure 8a). Dense accumulation of lipiodol with miriplatin was shown during B-TACE (arrow in Figure 8b,c). 

Experiment 2 (i.e., a single tumor and unsuccessful B-TACE) is similar to the case of a patient (patient 2) that underwent B-TACE after an early 1-cm tumor at S8 (i.e., the therapeutic target) was detected with MRI (Figure 8d). During embolization of S8, the microballoon tip pressure was above 64 mmHg, suggesting that collateral circulation existed. Indeed, accumulation of lipiodol with miriplatin was not enhanced (arrow in Figure 8e,f) as a pronounced collateral pathway emerged toward S4 (arrowhead in Figure 8e,f). These two clinical cases show that the IVM can reproduce clinically realistic scenarios.

## 3. Results

### 3.1. Experiments 1–4

**Figure 9:**
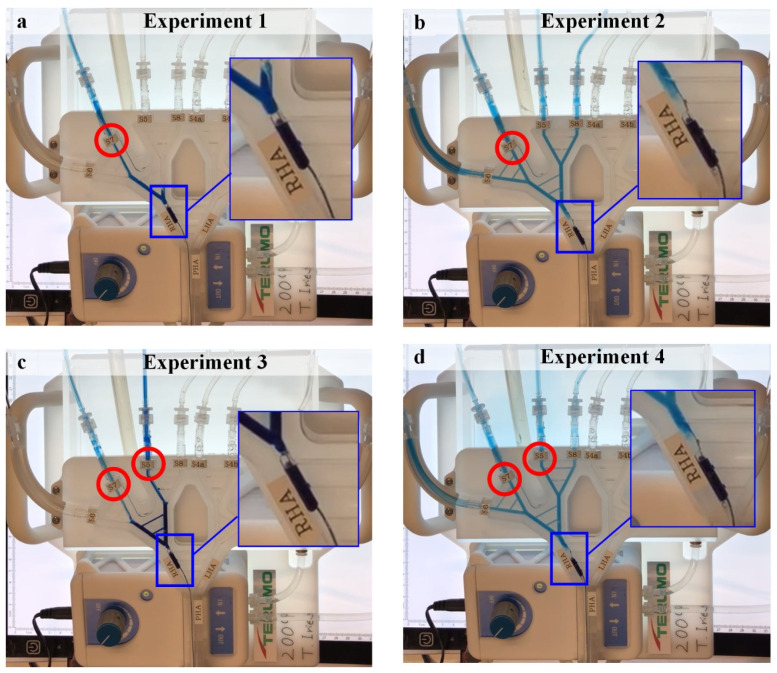
The results for Experiment 1–Experiment 4. (**a**) Experiment 1, (**b**) Experiment 2, (**c**) Experiment 3, and (**d**) Experiment 4. Dyed water mimics the injected solution, red circles indicate the tumor-bearing segments, and a detail of the relative position of the microballoon and the collateral arteries is shown. The videos of the experiments are available as Supplementary Materials.

The results of Experiments 1 to 4 with two user-controllable variables of two cancer scenarios and two microballoon catheter locations shown in Figure 9 match the predicted results in Figure 7. In the experiments where the microballoon is located distal to the pathways that model the CA, PGE is generated, and a tumor-directed flow is promoted (Experiments 1 and 3, Figure 9a,c). However, when the microballoon is between the pathways that model the CA, a collateral flow is promoted and the flow is in the direction of all the segments: both tumor and normal tissues in the model (Experiments 2 and 4, Figure 9b,d). The videos of the experiments are available as Supplementary Materials.

**Figure 10:**
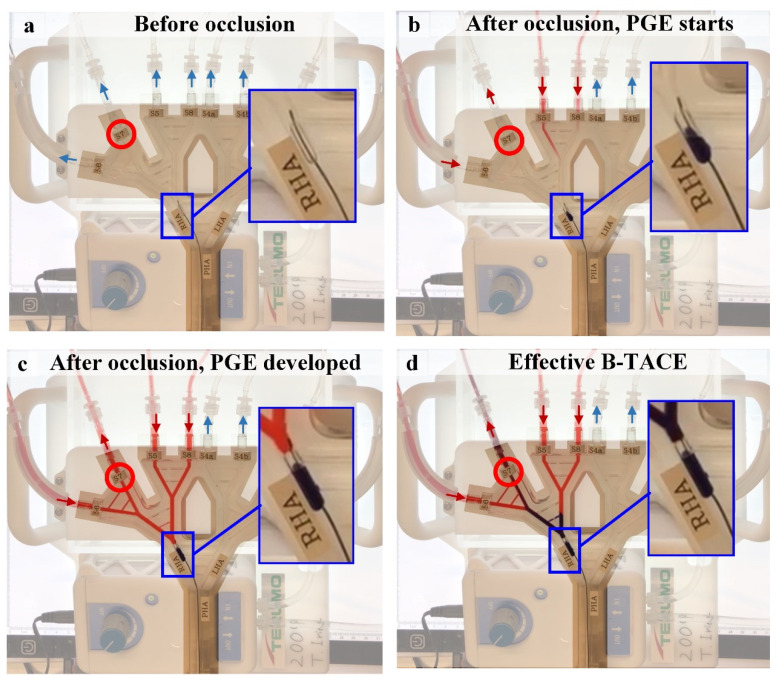
Snapshots of the video of Experiment 1 for the visualization of the PGE-generated flow. (**a**) Before occlusion, the flow is from the PHA to the segmental arteries. (**b**) When balloon occluding, PGE starts, and red dyed water starts to flow from the segmental arteries toward the RHA. (**c**) After a short period of time, a steady flow from the S6, S5, and S8 segmental arteries to the S7 segmental artery exists. (**d**) When injecting, the treatment is effective because the injection is directed toward the tumor-bearing segment. Red circles indicate the tumor-bearing segment and arrows indicate the direction of flow. The video of the experiment is available in the Supplementary Materials.

The normal use of the IVM was modified in order to perfectly visualize PGE. Experiments 1 and 2 were repeated with water with red ink in the upper reservoir, so that red ink would flow towards the hepatic artery if PGE were generated by balloon occlusion. Figure 10 shows several snapshots of the response of the system to the PGE-generating balloon occlusion in Experiment 1. The video of the experiment is available in the Supplementary Materials.

### 3.2. Experiment 5

**Figure 11:**
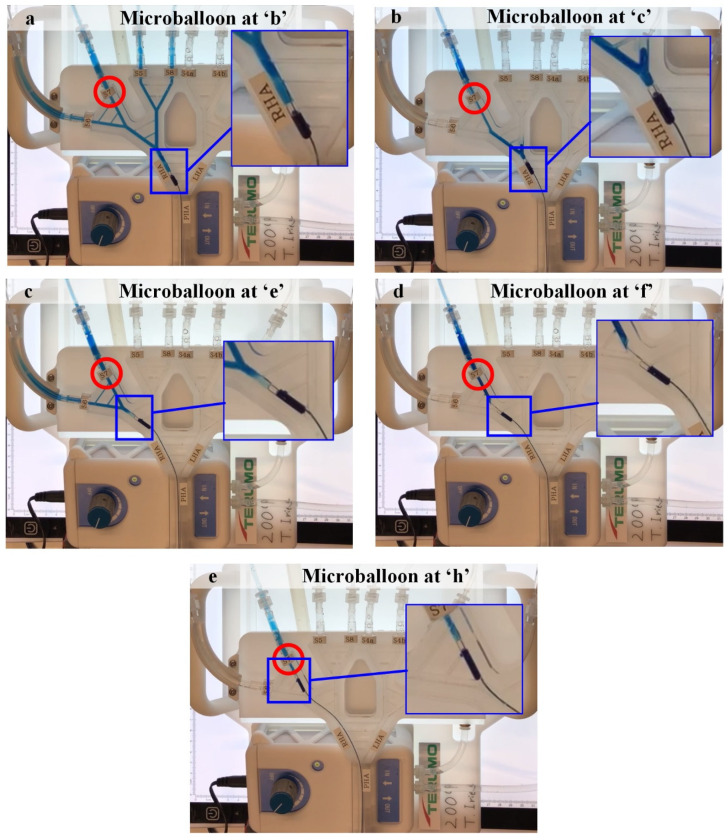
The results for Experiment 5. (**a**) Microballoon at ‘*b*’, (**b**) microballoon at ‘*c*’, (**c**) microballoon at ‘*e*’, (**d**) microballoon at ‘*f*’, and (**e**) microballoon at ‘*h*’. Dyed water mimics the injected solution, red circles indicate the tumor-bearing segments, and a detail of the relative position of the microballoon and the collateral arteries is shown. The videos of the experiments are available as Supplementary Materials.

Figure 11 shows the results for Experiment 5. In the in vitro experiment, only microballoon locations ‘*b*’, ‘*c*’, ‘*e*’, ‘*f*’, and ‘*h*’ were studied because of the length of the microballoon, making it impossible to study microballoon positions ‘*a*’, ‘*d*’, and ‘*g*’. According to the experiments, positions ‘*c*’, ‘*f*’, and ‘*h*’ result in effective B-TACE injections (i.e., directed to the tumor-bearing segment).

### 3.3. Numerical Simulations of Experiment 5

**Figure 12:**
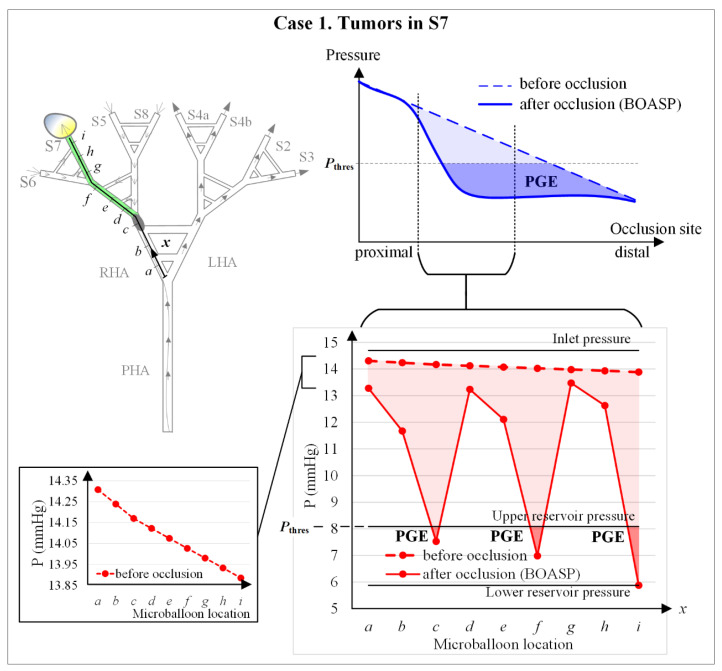
Numerical simulation results for Experiment 5: Case 1 (tumors in S7) with multiple microballoon locations (‘*a*’ to ‘*i*’). **Top left**: schematics of the hepatic artery with the tumor location and the multiple microballoon locations. **Top right**: conceptual *Pressure*–*Occlusion site* curves in real livers during B-TACE. The *x*-axis represents proximal to distal locations, while the *y*-axis represents the average arterial pressure before and after occlusion. **Bottom right**: *Pressure*–*Occlusion site* curves in the IVM during B-TACE. The IVM covers the central part of the top right figure, between proximal and distal occlusions. In addition, the inlet pressure and the upper and lower reservoir pressures are included. PGE is generated when the BOASP is between the upper and lower reservoir pressures. A detail of the pressure before occlusion is included on the **Bottom left**.

For this multiple-location experiment, numerical simulations were also conducted to obtain and discuss quantitative results of pressures and flows, and the results are shown in Figure 12. The top chart shows the physiologically realistic liver behavior during B-TACE for proximal to distal microballoon locations in the hepatic artery. Before occlusion or under normal conditions, the average blood pressure decreases linearly as the occlusion site moves from proximal to distal positions. After occlusion, the BOASP depends on the occlusion site, and is influenced by the collateral network surrounding the main hepatic artery tree. The bottom chart shows the results of the numerical simulations of the water flow in the IVM. Three important lines are included in the chart. 

The pressure at the inlet of the hepatic artery (IP), the pressure resulting from the height of the free surface of water in the upper reservoir (URP) and the pressure resulting from the height of the free surface of water in the lower reservoir (LRP). The pressure of the water in the in vitro hepatic artery will always be between IP and LRP. When there is no occlusion or before occlusion, the pressure decreases linearly, and most of the pressure loss takes place outside the in vitro hepatic artery in the tubes connecting the outlets of the hepatic artery and the reservoir. When occluding between ‘*a*’ and ‘*i*’, the collateral network modeled in the IVM defines the value of the BOASP, which generates PGE if the BOASP falls between the URP and the LRP. 

When the BOASP is greater than the URP, the water after the occlusion site has the power to flow towards both reservoirs. When the BOASP is smaller than the URP, the water after the occlusion site does not have the power to flow towards the reservoirs. Instead, the upper reservoir feeds the hepatic artery and a flow between the upper and lower reservoirs is promoted (see Figure 2). The URP can, therefore, be seen as the threshold pressure defined by Irie et al. [6] as 64 mmHg. 

This PGE-generating situation is only reached for occlusion sites ‘*c*’ and ‘*f*’ because of the simplicity of the collateral network modeled. As seen in experiments, occlusion sites ‘*c*’ and ‘*f*’ are locations with effective B-TACE treatment. Occlusion at ‘*h*’ is also effective, even if PGE is not generated, because of the smaller hydraulic resistance towards S7 compared with towards S6.

## 4. Discussion

Despite the efficacy of the newly developed therapeutic procedures, it is often difficult to explain the mechanism or to reproduce it in every different clinical scenario. Microballoon catheters have emerged as promising devices to reduce nontarget embolization during various intravascular procedures [17,30,31]. Specifically, in the case of B-TACE, the use of these devices has shown the potential to improve the outcome of conventional TACE [6,7,8,32]. The use of drug-eluting microspheres injected via a microballoon catheter has also been successfully explored [33]. 

When the normal circulation is altered by a temporal balloon occlusion in a main branch of the hepatic artery, collateral circulation is promoted as a result of the arterial connections between the upstream and downstream compartments [9] through collateral pathways in the liver, such as the interlobar CA [11,28], the arteries of the peribiliary plexus [34], and isolated arteries [35]. The vascularity of the organ is complex because the collateral circulation is not always visible prior to the treatment; some collateral circulation suddenly emerges during the embolization. 

Irie et al. [6] found PGE and reported a value for the BOASP below 64 mmHg as an indicator for a successful treatment. The conclusion suggested that PGE plays the important role in redistributing the vascular circulation and promotes the accumulation of the lipiodol emulsion in cancer tissues. Microballoon catheters and antireflux catheters were shown to provide safety because they prevent the retrograde treatment agent delivery and an increased efficiency because of the preferential targeting towards tumors [9]. Thus, microballoon interventions take advantage of the complex vascular circulation of the liver. The present study shows that the IVM, which models the arterial circulation in the liver, is a simple model that is able to replicate PGE.

Figure 12 shows the qualitative behavior of the pressure distal to the microballoon. In a physiologically realistic behavior of blood pressure in the hepatic artery, the pressure is expected to decrease linearly from proximal to distal locations with no balloon occlusion. During balloon occlusion, PGE emerges at a certain range of occlusion locations when the BOASP is low enough (top right chart of Figure 12). 

When occluding very proximally, the effect of balloon occluding is unnoticeable because the collaterality is very high. The pressure in the IVM in the numerical simulations of Experiment 5 decreases from proximal to distal locations with no balloon occlusion (the left bottom chart of Figure 12); and PGE is generated with balloon occlusion near ‘*c*’ and ‘*f*’, that is, when BOASP is below the URP, the equivalent to the threshold BOASP defined by Irie et al. [6] as 64 mmHg.

In the in vitro experiments of this study, neither very proximal nor very distal microballoon locations were examined (see the connection between the top right and bottom right charts in Figure 12). If blood pressures after occlusion in the physiologically realistic behavior and the IVM behavior are compared, PGE is only generated when occluding near ‘*c*’ and ‘*f*’ in the IVM. This difference is due to the fact that the collaterality in the IVM is represented via the collaterals in the in vitro hepatic artery, while the collateral network existing in the liver is much more complex [13]. 

However, although a simple hepatic artery model generates simple, limited scenarios of collateral circulation and flow redistribution triggered by PGE, the IVM allows for the visualization of the seemingly invisible collateral circulation by means of a gravitation-driven hemodynamic phenomenon that represents liver parenchyma at the upper reservoir and tumor tissues at the lower reservoir’s water free surface at different heights.

The IVM allows for the simulation of certain clinically meaningful scenarios by selecting the tumor-bearing or normal segments and the microballoon locations, due to the simplicity of the model. For instance, PGE at the location ‘*c*’ and ‘*f*’ represents the scenario of the collateral pathway at hilar plate (RHA to/from LHA). However, the PGE phenomenon can also occur at the micro level in small vascular networks (e.g., in the peribiliary plexus) and this IVM can be used to visualize and understand what occurs at the micro level during B-TACE. 

In fact, Experiments 1 and 2 are similar to the clinical cases shown in Section 2.5 as patients 1 and 2, respectively. Therefore, we could say that, if these experiments represent clinical cases, then the conceptual IVM design was appropriate in the first place. Despite the value of the IVM, the current version of the IVM has three limitations to study in the future.

First, the use of water as the working fluid limits the pressure values in the hepatic artery of the IVM. These pressures are not physiologically realistic because the viscosity of water differs from that of blood. To overcome these limitations, a proper water–glycol mixture resulting in a density of 1050 kg/m^3^ and a viscosity of 0.0035 Pa·s will be used to better simulate blood. 

Second, a representative hepatic artery geometry applied idealized collateral vessels between hepatic artery branches excluding the portal vein system. This also relates to the fact that only two pressure levels are defined in the system: one for the liver parenchyma and the other for tumor tissue. This limits all the hepatic artery branches feeding both liver parenchyma and tumors are at the same pressure level, meaning that the intratumoral vascular resistance is considered equal for all tumor nodules. 

Third, the IVM could not replicate tumor tissue accumulation by embolic agents. For future investigation of the model development based on computational fluid dynamics or artificial intelligence-based calculation and simulation of hemodynamics, more challenging hepatic artery geometries will be created together with multiple pressure levels and different types of embolic agents, so that the IVM enables us to simulate patient-specific cases in various treatment options.

## 5. Conclusions

In conclusion, the computationally designed IVM, despite its simplicity, can help visualize and understand the arterial hemodynamics in normal conditions and the complex PGE during B-TACE. The IVM could be a valid educational tool for medical training in B-TACE and it could serve as a base for more sophisticated, even patient-specific, IVM models to be used as a research tool.

## Figures and Tables

**Table 1:** Parts of the IVM and its function, size, and material.

# (Figure 6)	Part Name	Function	Material
1	Irrigation pump	To reproduce the arterial mean pressure that drives flow. Flowrate: 0–500 mL/min. Power: AC100 V	
2	Hepatic artery model	To reproduce the real-scale hepatic artery flow to perform in vitro simulations of B-TACE.	Acrylic polymer
3	Liver flow dock and panel	To firmly locate the hepatic artery model in the desired height	Acrylic polymer
4	Upper reservoir	To replicate normal tissue of liver.	Acrylic polymer
5	Lower reservoir	To replicate tumor tissue. It also provides fluid with pooling space.	Acrylic polymer
6	Pump reservoir	To replicate vein’s outflow and feed the pump.	Acrylic polymer
7	Outflow drain	To keep the free surface level of water at the desired height.	Silicon
8	Three-way stopcocks	To permit flow toward upper or lower reservoir.	Poly propylene
9	Liver flow outlet tubes	To connect the hepatic artery model to the upper/lower reservoir.	Polyvinylchloride
10	Inflow tube	To replicate celiac artery, common hepatic artery and proper hepatic artery to allow the catheter systems to deliver access the occlusion site.	Polyvinylchloride
11	Outflow tubes	To circulate fluid in lower reservoir to irrigation pump	Polyvinylchloride

## Data Availability

Data and codes are available under request to the authors.

## Supplementary Materials

The following are available online at https://www.mdpi.com/article/10.3390/biology10121341/s1, Video S1: Video of Experiment 1. Video S2: Video of Experiment 2. Video S3: Video of Experiment 3. Video S4: Video of Experiment 4. Video S5: Video of Experiment 5 with microballoon at ‘*c*’. Video S6: Video of Experiment 5 with microballoon at ‘*e*’. Video S7: Video of Experiment 5 with microballoon at ‘*f*’. Video S8: Video of Experiment 5 with microballoon at ‘*h*’. Video S9: Video of Experiments 1 and 2, where PGE development is clearly visualized.
